# Mesoscale explorer: Visual exploration of large‐scale molecular models

**DOI:** 10.1002/pro.5177

**Published:** 2024-09-18

**Authors:** Alexander Rose, David Sehnal, David S. Goodsell, Ludovic Autin

**Affiliations:** ^1^ Independent San Diego California USA; ^2^ National Centre for Biomolecular Research, Faculty of Science Masaryk University Brno Czech Republic; ^3^ Department of Integrative Structural and Computational Biology The Scripps Research Institute La Jolla California USA; ^4^ Research Collaboratory for Structural Bioinformatics Protein Data Bank, Rutgers The State University of New Jersey Piscataway New Jersey USA

**Keywords:** 3D animation, interactive tours, mesoscale models, molecular graphism, web‐based 3D visualization

## Abstract

The advent of cryo‐electron microscopy (cryo‐EM) and cryo‐electron tomography (cryo‐ET), coupled with computational modeling, has enabled the creation of integrative 3D models of viruses, bacteria, and cellular organelles. These models, composed of thousands of macromolecules and billions of atoms, have historically posed significant challenges for manipulation and visualization without specialized molecular graphics tools and hardware. With the recent advancements in GPU rendering power and web browser capabilities, it is now feasible to render interactively large molecular scenes directly on the web. In this work, we introduce *Mesoscale Explorer*, a web application built using the Mol* framework, dedicated to the visualization of large‐scale molecular models ranging from viruses to cell organelles. *Mesoscale Explorer* provides unprecedented access and insight into the molecular fabric of life, enhancing perception, streamlining exploration, and simplifying visualization of diverse data types, showcasing the intricate details of these models with unparalleled clarity.

## 1 INTRODUCTION

In 1966, the first 16 mm movies of animated proteins were produced by C. Levinthal (Levinthal, 1966). This pioneering work was followed by a movie of the Insulin Dimer made by the *Molecular Modeling System* (Marshall et al., 1972) (MMS) devised by J. J. Beitch, R. A. Ellis, J. M. Fritch and G. R. Marshall. Since then, technological advancements have continually enhanced tools for interactively manipulating proteins and generating explanatory animations and movies (McGill, 2008; Riggi et al., 2024).

Several interactive molecular viewers have been developed over the years, including *GRAMPs* (O'Donnell & Olson, 1981), *Kineimage* (Richardson & Richardson, 1992), *rasmol* (Sayle, 1995), *VMD* (Humphrey et al., 1996), *Ribbon* (Carson, 1997), *PMV* (Sanner, 1999), *Pymol* (Delano, 2002), *BallView* (Moll et al., 2005), *Chimera* (Pettersen et al., 2004; Pettersen et al., 2021), and *YASARA* (Ozvoldik et al., 2023). The advent of the internet has further democratized molecular structure manipulation with web‐based viewers like *Jmol* (Anon, n.d.), *NGL* (Rose et al., 2018), *LiteMol* (Sehnal et al., 2017) and *iCn3D* (Wang et al., 2020). As interactivity became more accessible, additional features for animation and movie making were developed in desktop applications (e.g., *YASARA* movies [https://yasara.org/movies.htm], *eMovie* [Hodis et al., 2007], *ChimeraX* movie [Goddard, 2017]) as well as plugins for general 3d animation software (*mMaya* [https://clarafi.com/tools/mmaya/], *ePMV* [Johnson et al., 2011], *Molecular Nodes* [https://bradyajohnston.github.io/MolecularNodes/]) and web platforms (*PolyviewMM* [Porollo & Meller, 2010], *movieMaker* [Maiti et al., 2005], *PMG* [Autin & Tufféry, 2007], *activeICM* [Raush et al., 2009]). This evolution led to the creation of outreach platforms such as *Proteopedia* (https://proteopedia.org), *FirstGlance* in *Jmol* (https://www.bioinformatics.org/firstglance/fgij/), and *Jolecule* (https://jolecule.appspot.com/), which provide guided macromolecular visualization with ease of use.

However, these platforms often struggle with the increasing complexity and size of contemporary molecular models, particularly with the advent of the “resolution revolution” in cryo‐electron microscopy (Kühlbrandt, 2014) (cryo‐EM and cryo‐ET) and multiscale molecular modeling techniques such as *cellPACK* (Johnson et al., 2015; Klein et al., 2018), *YASARA* PETWORLD (Ozvoldik et al., 2021), and *Mesocraft* (Nguyen et al., 2021). These advances have enabled the creation of comprehensive 3D models of viruses, bacteria, and cellular organelles, comprising thousands of macromolecules and billions of atoms. The widespread availability of these models has opened new development avenues for their exploration (Alharbi et al., 2023; Kadir et al., 2021; Kouril et al., 2023) and rendering (Alharbi et al., 2024; Falk et al., 2013; Le Muzic et al., 2015; Lindow et al., 2012). New ways to visualize molecular structures in their cellular context are emerging, ranging from guided tours in Apple Vision Pro with *cellWalk* (https://cellwalk.ca/) to entire educational courses built around mesoscale animations in *SmartBiology* (https://www.smart-biology.com). It is crucial that these advancements are accessible to a broad audience, not limited by specific hardware or software.

In alignment with these technological strides, we present the *Mesoscale Explorer*, an innovative web application designed for exploring large molecular models, spanning from viruses to cell organelles. Primarily targeted at researchers, educators, and students in molecular and structural biology, it offers an interactive experience with guided tours and animations to navigate the complexities of these models. These guided tours are also accessible to a wider general public, providing an opportunity for anyone interested to gain unprecedented access and insight into the molecular fabric of life. Built upon the robust open source Mol* framework (Sehnal et al., 2021), *Mesoscale Explorer* leverages cutting‐edge web technologies to render intricate details of large‐scale models consisting of billions of atoms.

Applications like *Mesoscale Explorer* are at the forefront of the effort to democratize molecular and cellular knowledge, offering intuitive interfaces and guided explorations that demystify the molecular universe. Accessible online at https://molstar.org/me/, the *Mesoscale Explorer* invites users to delve into the world of molecular landscape visualization. The dedicated landing page offers links to directly access and explore various models and guided tours within the viewer. Additionally, comprehensive documentation and tutorials are available at https://molstar.org/me-docs/, designed to help users maximize their experience with the *Mesoscale Explorer*, ensuring a smooth and enriching journey through the molecular landscapes that underpin life itself.

## 2 METHODS

*Mesoscale Explorer* is built on top of Mol*, a cutting‐edge library designed for developing web applications to visualize molecular data. This initiative, born from a collaboration between *PDBe* and *RCSB PDB* (the European, American branches of the Worldwide Protein Data Bank; https://www.wwpdb.org) and *CEITEC* (https://www.ceitec.eu/), aims to synergize the capabilities of *LiteMol* (Sehnal et al., 2017) (developed by PDBe [Varadi et al., 2022]) and *NGL* (Rose et al., 2018; developed by RCSB PDB [Berman, 2000]) viewers. Mol* stands out as a custom rendering engine optimized for molecular graphics on the web and is currently the default interactive viewer at the *RCSB PDB* (www.rcsb.org). In this work, we extend Mol* to support real‐time rendering of molecular scenes encompassing billions of atoms. To enhance performance and tackle common bottlenecks associated with rendering large‐scale scenes, we developed several optimizations inspired by the state of the art implementation introduced by *cellVIEW* (Le Muzic et al., 2015).

### 2.1 Supported models

Our intention is to support models coming from any available modeling and experimental methods that provide data in a compatible format. We have currently limited our supported format to mmCIF and explored a new generic container, known as a manifest. This manifest, implemented as a ZIP archive, embeds a JSON file that organizes metadata and references associated binary files, allowing for flexible and structured data storage.

- mmCIF and BinaryCIF: The macromolecular Crystallographic Information File (mmCIF) format and its binary version (Sehnal et al., 2020) developed by the wwPDB (Berman, 2000) has become the standard in the structural biology domain. This format augments the CIF (Crystallographic Information File) format by intricately detailing the complexity inherent to biological macromolecules, including proteins, nucleic acids, and their complex assemblies. Structured as a text file, mmCIF organizes information into data blocks filled with tagged items, making it intelligible to both humans and computational tools. For expansive models, it employs Biological Assembly Descriptions (e.g., _pdbx_struct_assembly, _pdbx_struct_assembly_gen, _pdbx_struct_assembly_prop, _pdbx_struct_oper_list) to spatially arrange molecule instances within three‐dimensional spaces, linking these assemblies to specific protein chains of the entities described. We introduce an optional customization feature, allowing the incorporation of comprehensive markdown descriptions for every entity within the mmCIF schema. Furthermore, our system accommodates the specialized format of *YASARA* Pet‐World models (Ozvoldik et al., 2021), which is an adapted version of mmCIF. These files encapsulate multiple, distinct objects, delineated by progressively increasing atom_site.pdbx_PDB_model_num. The first pdbx_struct_assembly stores their locations, while a *YASARA*‐specific extension appends the model number (pdbx_struct_assembly_gen.PDB_model_num) and annotates object names (pdbx_model.name) along with instance numbers (pdbx_model.instances). Our platform also supports markdown descriptions within the *YASARA* mmCIF files, provided they are manually included in the designated fields.
- Manifest Files: In response to the need for enhanced efficiency in processing generic mesoscale models, we have developed a container format with a manifest file. This format is predicated on a JSON‐based structure that meticulously outlines the scene, incorporating a group filter, a comprehensive list of entities (in binary cif format), their group attributes, and spatial information (position and rotation) stored in streamlined binary files. This approach is designed to significantly reduce storage demands while maintaining precision and fidelity in model representation and interaction.
- Mol* session: These files are a dedicated Mol* format that captures states and assets of a given session. This format is used to store guided tours and pre‐made scenes.

### 2.2 Reducing rendering costs

Real time rendering of billions of atoms is achieved by: (1) taking advantage of patterns in mesoscale models and (2) applying graphics techniques for rendering large scenes from, for example, video games. Our approach includes (a) use of instancing to draw multiple copies of identical structures, (b) representation of structures with simple sphere geometries, (c) reduced detail based on the distance to the camera, and (d) drawing of only what is potentially visible, that is, not covered and within the camera view. Additionally, we can decrease the rendering resolution and approximate sphere geometries.

For mesoscale models, we assume that all structures of molecular entities of the same type are generally identical. For example, all copies of hexokinase in a cytoplasm model are assumed to have the same structure, meaning they share the same general atomic coordinates. This assumption enables the use of a very fast instancing approach for visualization. Only a single set of atomic coordinates is required for each molecular entity, which can be passed to the GPU once and then used to render all instances of that entity in the mesoscale model by applying their individual positions and rotations. We use spheres to represent structures because they are a common, familiar type of representation; they can be rendered very efficiently with high quality using ray‐casted impostors (Grottel et al., 2009; Gumhold, 2003) and they allow easy simplification by combining multiple adjacent spheres into a single sphere to reduce visual clutter and rendering cost. As structures move further from the camera, reducing their visual detail allows us to scale from virus capsid models to representations of small bacteria or cell organelles at a high frame rate. Since mesoscale models are generally densely packed, we can cull (i.e., not draw) structures that are not within the camera (e.g., when zoomed‐in to part of the model) or completely covered by nearer structures using frustum or occlusion culling, respectively.

**FIGURE 1:**
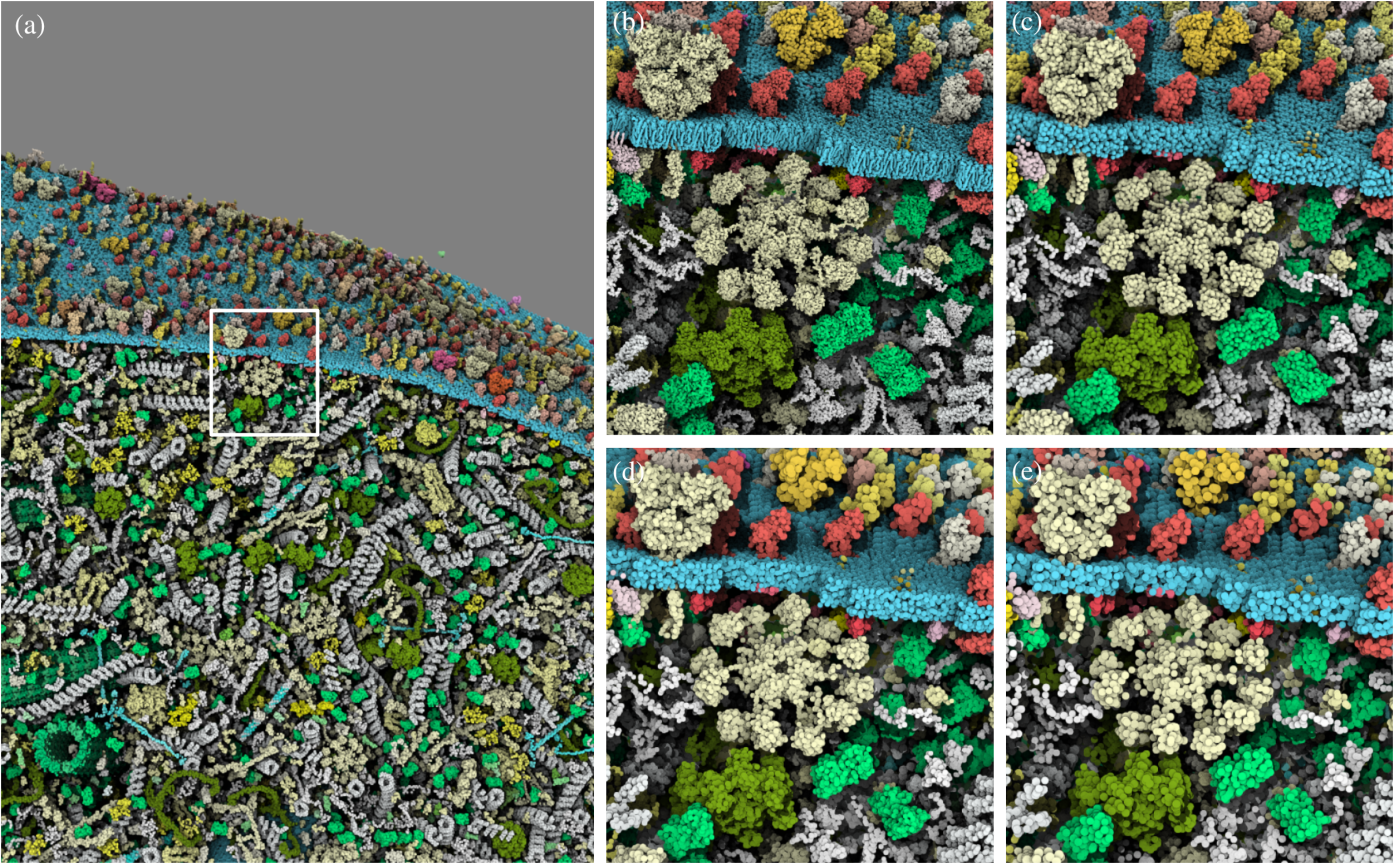
User‐definable options for quality of rendering with closeup zoom: (a) Default quality large view of the model with a white square selection for close‐up view: (b) highly detailed but costly Ultra option, (c) default detailed rendering Quality mode, (d) medium rendering quality rendering with approximate sphere Balanced mode and (e) the lower resolution and highly interactive Performance option. Explore this interactively.

Variable graphical preset modes are available to suit the user's needs and hardware capabilities. Level of detail (LOD), resolution, and sphere approximation (e.g., flat disc option) are the parameters that vary per mode. As illustrated in Figure 1, we currently provide four modes, sorted by decreasing resolution and details, that enable increasingly higher framerate. The Ultra mode provides the most detailed view of the model by keeping more atomistic details with very high LOD; the default Quality mode provides the best compromise between atomic details with high LOD and framerate; the Balanced modes uses sphere approximation and medium LOD; the Performance mode focuses on high frame rate at the price of a lower resolution with low LOD.

### 2.3 Enhancing perception

Visual perception in molecular rendering can be improved by refining the lighting and shadowing techniques employed. Shadowing can clarify complex 3D spatial relationships and topologies, but traditional shadow rendering often necessitates the computationally intensive task of redrawing the entire scene from the light source's viewpoint. To circumvent this challenge and streamline the rendering process, we have adopted a screen space shadow technique (Aldridge, 2023; Karabelas, 2020), also known as contact shadowing. This method calculates shadows directly within the screen space by tracing a path from each pixel toward the light source. At each step along this path, we evaluate the depth of our ray against the depth perceived by the camera. If the ray's depth exceeds that of the camera's view, it implies that the pixel lies in shadow.

**FIGURE 2:**
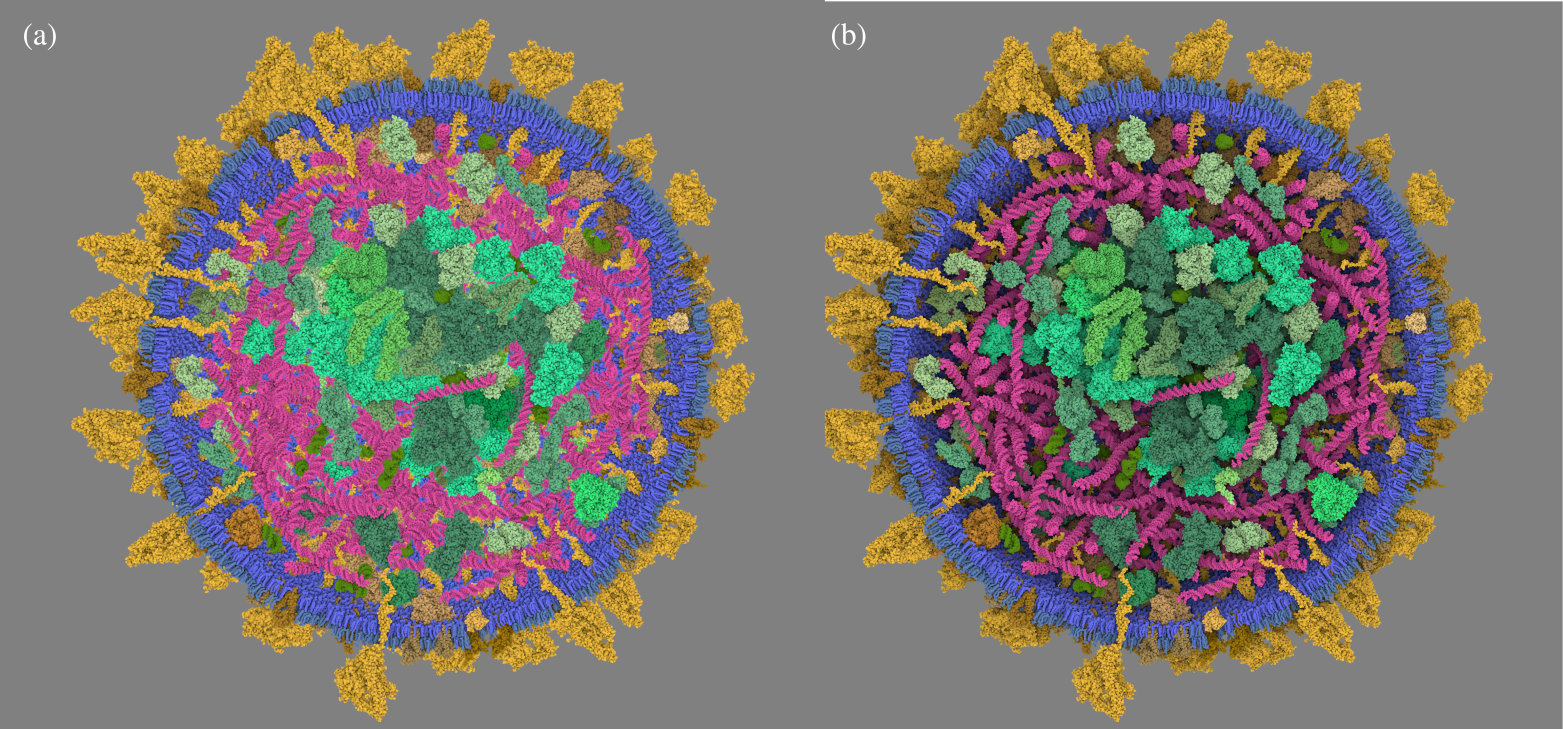
Illustration of the SSAO post‐process. (a) On the left, standard SSAO that emphasizes local occlusion. (b) On the right, our multiscale approach that captures occlusion at a farther distance. See it interactively at this link.

We complement this shadowing technique with Screen Space Ambient Occlusion (SSAO), a revered technique in computer graphics designed to approximate the ambient occlusion effect in real‐time. SSAO enhances the depth and realism of 3D scenes by mimicking the way light radiates in real‐world environments, particularly how it struggles to penetrate tight spaces. Given the unique demands of molecular landscapes, we tailor our SSAO implementation to suit. Based on the state‐of‐the‐art method from Filion and McNaughton (Filion & McNaughton, 2008), our customized approach varies the occlusion radius across multiple steps, allowing for a nuanced distinction between close‐contact atoms and the broader spatial relationships among molecular chains or protein complexes. As illustrated in Figure 2, this dual strategy of tailored shadowing and SSAO aids in the visual differentiation of structural details, facilitating a deeper understanding of complex molecular formations, such as the nuanced interior of a virus capsid.

By leveraging these advanced lighting techniques, our goal is to not only improve the aesthetic quality of our molecular renderings but also enhance the user's ability to perceive and interpret intricate molecular structures with greater clarity and insight.

### 2.4 Clipping

**FIGURE 3:**
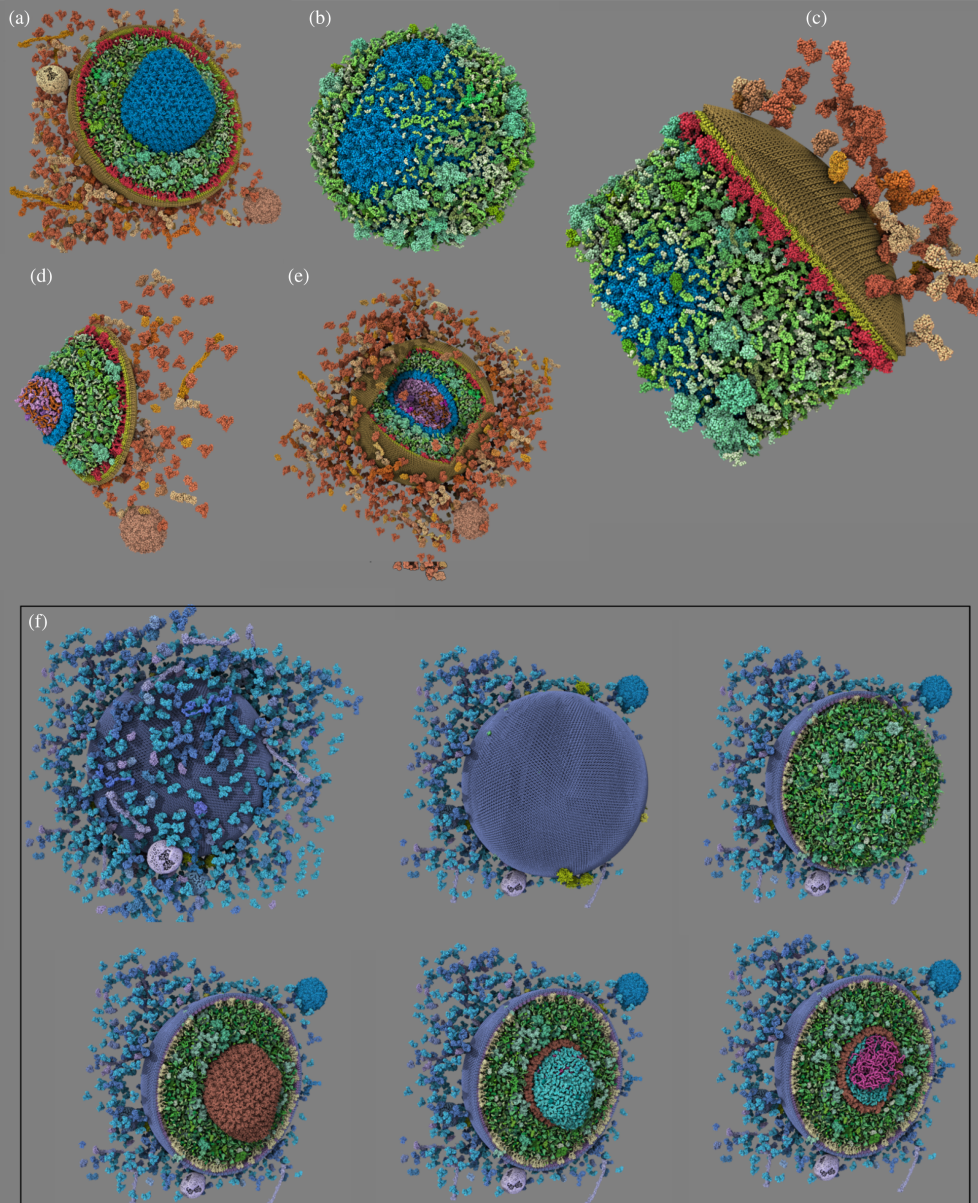
Clipping objects. (a) plane, (b) sphere (inverted), (c) cylinder (inverted), (d) infinite cone (inverted), and (e) cube. (f) Illustrate how we can use progressive clipping to reveal the different features of the HIV structural model, peeling layer by layer to reveal the genome inside. Explore this interactively.

To optimize the interactive visualization and exploration of large molecular landscapes, we have deployed sophisticated clipping strategies (see Figure 3). These strategies enable precise control over the parts of the molecular assembly that are visible within the viewport, effectively managing the rendering workload. Our toolkit supports an array of clipping objects based on well‐known distance functions and their usage (Quilez, 2008), including planes, spheres, cubes, cylinders, and infinite cones, offering a versatile range of geometric constraints for visual exploration.

The power of these clipping mechanisms lies in their selective applicability to distinct subsets of molecular entities. This targeted approach grants researchers the capability to tailor their visual analysis, enabling the crafting of complex visual narratives within the molecular landscape. For example, in Figure 3f, a virus model is dissected by clipping planes to expose its internal structure, while simultaneously ensuring that specific elements, such as its genetic material or capsid, remain highlighted and fully visible. This level of detail and flexibility not only enriches the visual representation but also deepens the analytical value of the rendered scenes, allowing for a more nuanced exploration of molecular structures.

**FIGURE 4:**
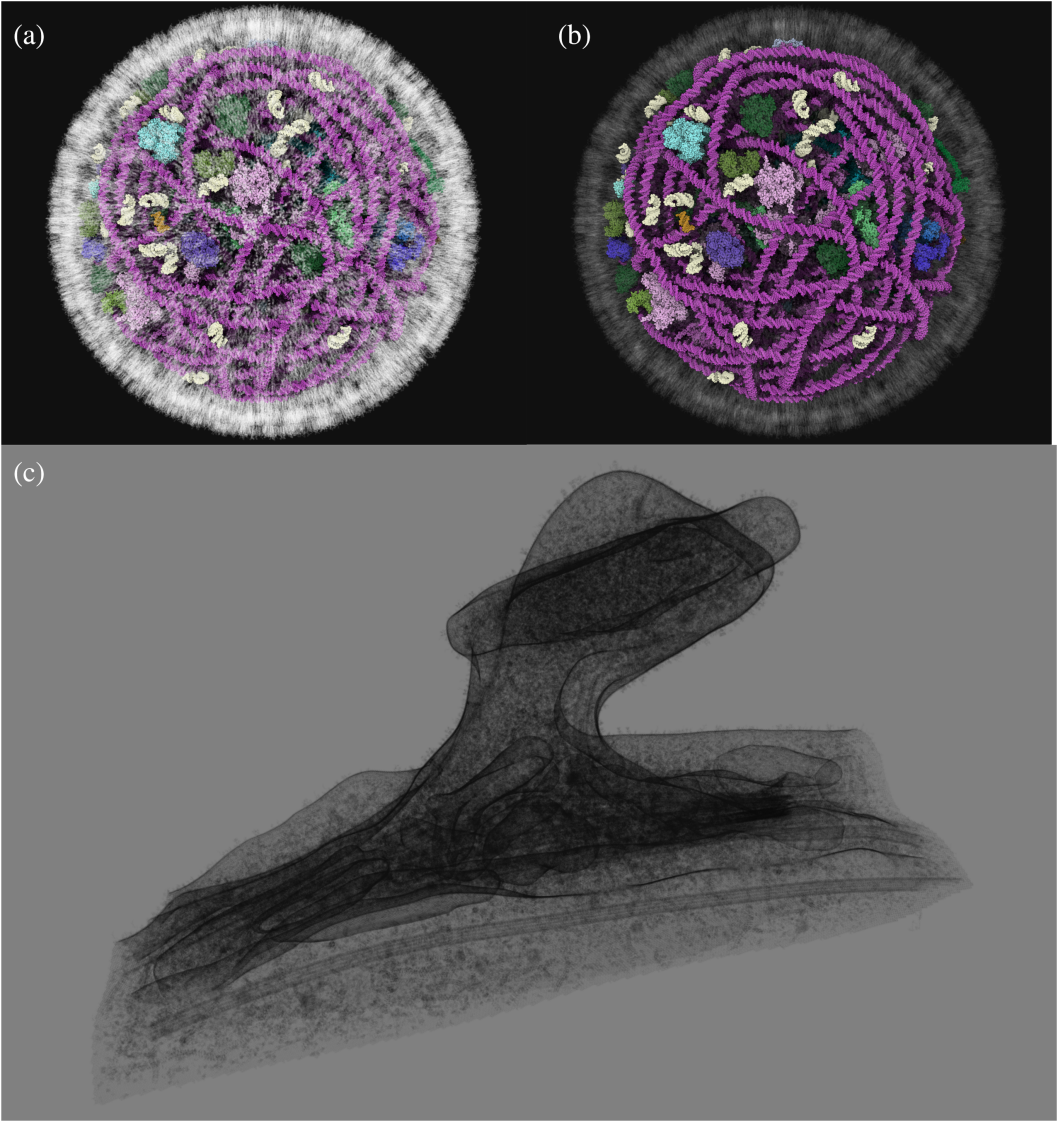
Transparent material. (a, b) Applied to the Exosome lipid membrane with two different opacity values (left 0.6 and right 0.1). Explore it interactively. https://molstar.org/me/viewer/?url=https://mesoscope.scripps.edu/explorer/examples/Figure4AB.molx&type=molx&hide‐controls=1. (c) Transparency (0.015) and black uniform color applied to the whole post‐synapse model, revealing its internal structure. Explore this interactively.

Enhancing the visibility of specific protein groups within a model can also be achieved through strategic use of transparency. However, the challenge lies in maintaining visual clarity when the rendered elements are exclusively spheres. Addressing this, we have designed a custom transparency shading to dynamically adjust the transparency, or alpha value, of each sphere based on its normal's orientation relative to the camera. This means that a sphere becomes more transparent when its normal points away from the user's perspective. This nuanced approach ensures that transparency not only serves its functional role in altering visibility but also contributes to the overall aesthetic quality of the rendering as illustrated in Figure 4.

### 2.5 Exploration

Mol* provides a large range of functionalities that enable users to delve into molecular models via both guided and user‐built tours and animations. At the heart of this feature are two foundational Mol* modules: snapshots and labeling. Snapshots capture the current state of the viewer and all the parameters currently in use (camera position, clipping, colors, visibility, etc.) stored together with a name, key and description. Labels are 3D text that can be added to any selection. Multiple snapshots can be created at any given moment and re‐played akin to a slideshow. Notably, Mol* facilitates automatic view interpolation between snapshots, adding a seamless, narrative flow to the presentation. This capability empowers users to weave a story through scenes that highlight and elaborate on significant areas within the models.

**FIGURE 5:**
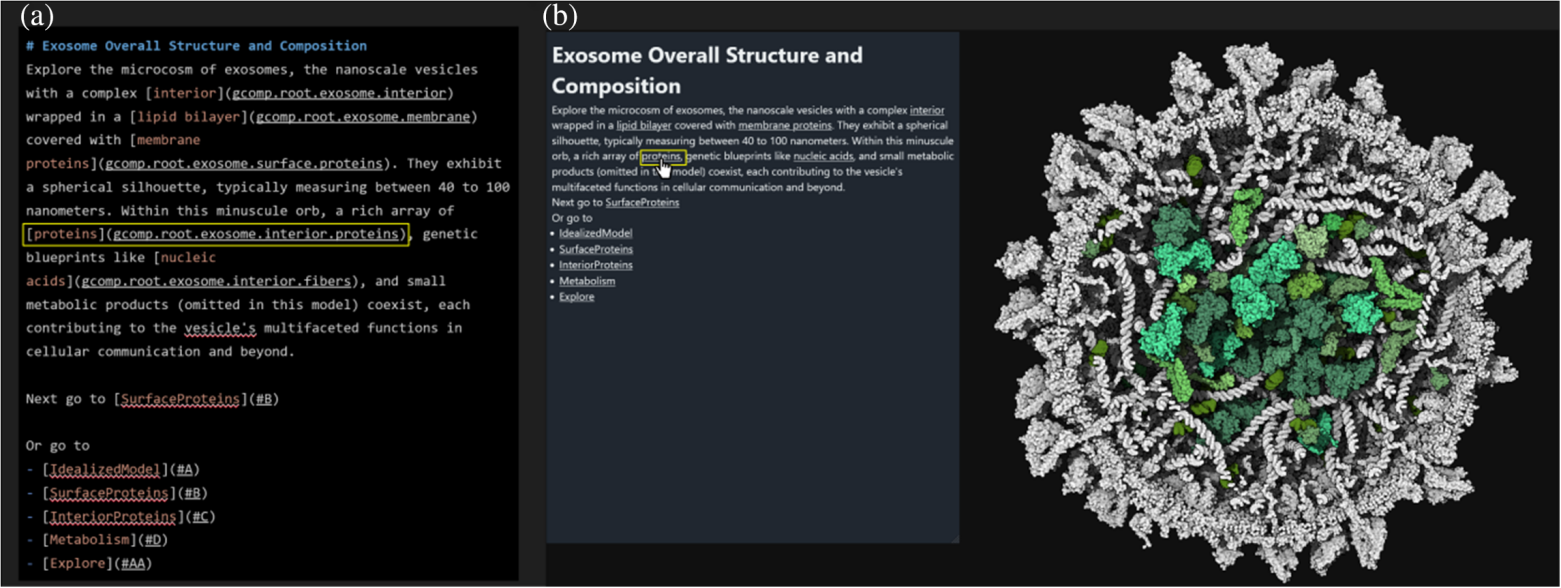
Markdown description. (a) Raw description utilizing Markdown syntax to include link to other snapshots as well as mouse hover highlighting feature. (b) Html rendering of the description and illustration of the mouse hover effect, the mouse is hovering the word ‘protein’ which highlights in the 3d view the interior protein, here in green color, while the rest of the model remains uncolored. Explore this interactively.

The storytelling is enriched through key bindings and Markdown (Gruber, 2004) annotations available in the snapshot description and labels, providing a layered, descriptive narrative over the visualization. Markdown is a lightweight markup language with plain‐text formatting syntax. In Markdown, the link text is enclosed in square brackets []. This is the visible text that the reader will click on. The target URL is enclosed in parentheses (). We currently support additional target links for snapshots with a given key, highlighting proteins with a given name, highlighting groups, thereby offering an interactive and immersive exploration tool. Figure 5 illustrates the rendering of one snapshot description (Figure 5a) in a widget viewport and the effect of the mouse hovering on the word ‘proteins’ (Figure 5b) that will highlight all the interior proteins of the model.

To further enrich the visualization, 3D labels serve as a powerful tool for annotating selected elements within a scene. These labels, when incorporated into the scene, are not just static markers; they are fully interactive and can be linked directly to snapshot keys. This feature significantly enriches the user experience by making the navigation through molecular tours more intuitive and captivating. Customization includes the label's text, color, a markdown tooltip for additional information that appears on mouse hover, a reference to a snapshot key that makes the label interactable in the viewer for a quick navigation to the specified snapshot, font style, border appearance, and tethering mechanism. This array of options empowers users to create a personalized and informative visual exploration environment. A complete tutorial on how to make a guided tour is available in the documentation page https://molstar.org/me-docs/tutorial/.

### 2.6 Guided tour

We have created tours for many currently published and accessible mesoscale models, including but not limited to the HIV mature virus (Johnson et al., 2014; Ozvoldik et al., 2021), Mycoplasma genitalium (Maritan et al., 2022), and Exosomes (Jiménez et al., 2019). A consistent methodology was applied across all tours to ensure a cohesive educational experience. Each journey begins with an introductory snapshot that presents the model's complete structure. Subsequent snapshots delve deeper, crafting a narrative that highlights features like membrane, critical proteins or biological process, guiding users from the general architecture down to the molecular intricacies.

As users navigate the model, interactive elements come to the forefront. A hover‐over feature activates when the mouse cursor moves over individual proteins, dynamically highlighting them and displaying their names in a tooltip window positioned at the bottom of the screen. If a detailed description is available within the model file, this information is also shown, providing valuable context and insights.

The textual content enriching these tours is the product of extensive research, drawn from a wealth of resources including literature reviews, the Protein Data Bank (Berman, 2000) (*PDB*), *UniProt* (UniProt Consortium, 2023), *KEGG* (Kanehisa et al., 2023), and *PDB‐101* (Zardecki et al., 2022). This information ensures that each tour is grounded in accurate and up‐to‐date scientific knowledge, offering a rich, immersive learning experience that spans the breadth and depth of mesoscale molecular biology.

## 3 RESULTS & DISCUSSION

**FIGURE 6:**
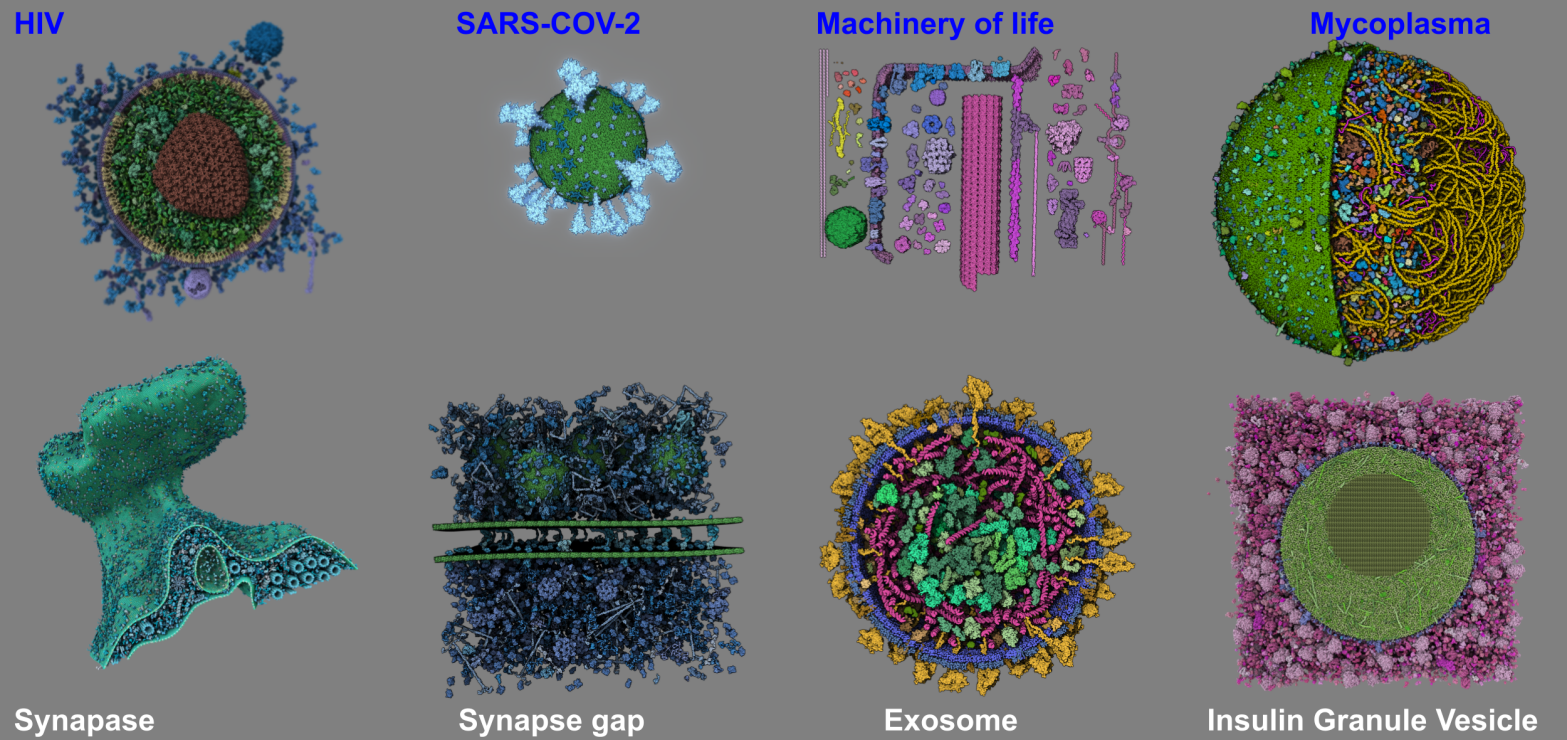
Examples of some of the growing number of available models in *Mesoscale Explorer* https://molstar.org/me/. Models with names in blue have guided tours available.

To illustrate performance and capabilities of the *Mesoscale Explorer* we provide a landing page with examples of standalone models as well as guided tours. In the sections below, we use standalone models to benchmark the viewer performance, and several guided tours to illustrate usage of the app. Figure 6 includes some of the currently available models and their corresponding tours, as more fully described in the documentation (https://molstar.org/me-docs/).

### 3.1 Model performance

**FIGURE 7:**
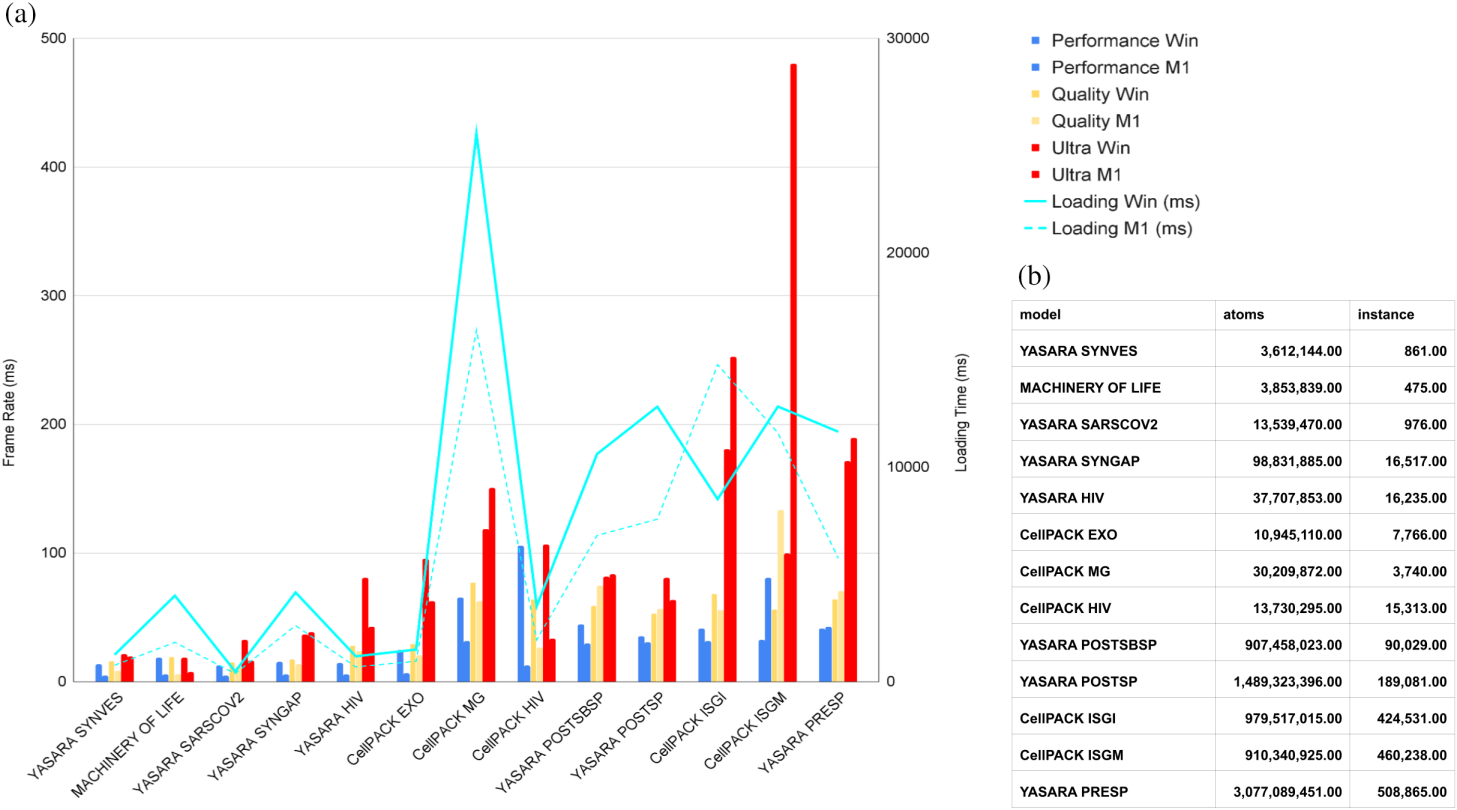
Average Performance. (a) Histogram of GPU rendering performance (left vertical axis in ms) combined with loading time (right vertical axis in ms) for various example models. The measurements were taken from the default view after loading each model and applying the rock animation. The x‐axis shows the model names, and the y‐axis displays the performance (lower values indicate better performance). Each color represents a quality setting (Quality, Performance, Ultra) and a machine (Windows, *Win*, with AMD Threadripper 1950X and Quadro RTX 8000 or Mac OS with apple silicon *M1*) at a resolution of 1598 × 1776. (b) Table of number of atoms and instances for each mode.

To evaluate the rendering performance of *Mesoscale Explorer*, we conducted a series of tests on models with different complexity. Figure 7 presents a histogram of GPU rendering performance, measured in milliseconds (ms), for each model. Lower values on the y‐axis indicate better rendering performance. The default ‘Quality’ setting emerged as the best compromise between performance and visual quality, achieving interactive performance for all models, even those with up to 3 billion atoms. Rendering performance was measured from a default view, which maintains a consistent distance from the model, as rendering performance is dependent on the camera's proximity. The Level of Detail (LOD) feature ensures that performance improves as the camera moves further from the object, while close‐up views are more demanding on the GPU.

### 3.2 Tours overview

Starting from a storyboard draft, the time required to produce a tour is approximately 2 h. The time needed to acquire the necessary information is not easily quantifiable, as it represents the culmination of years of research. However, the process of gathering and summarizing this information is streamlined through web‐based interfaces like *UniProt* (UniProt Consortium, 2023), as well as pre‐existing materials from *PDB‐101* (Goodsell et al., 2015; Zardecki et al., 2022) and *Wikipedia*. While we have attempted to utilize current Large Language Models (LLMs) to enhance our research, the results have been suboptimal. The information provided currently by LLMs often lacks sufficient detail, contains excess information, or includes inaccuracies and requires manual curation. Below we will describe some of the tours we developed to highlight some useful features offered by Mesoscale Explorer.

#### 3.2.1 Virus: SARS‐COV‐2 and HIV

**FIGURE 8:**
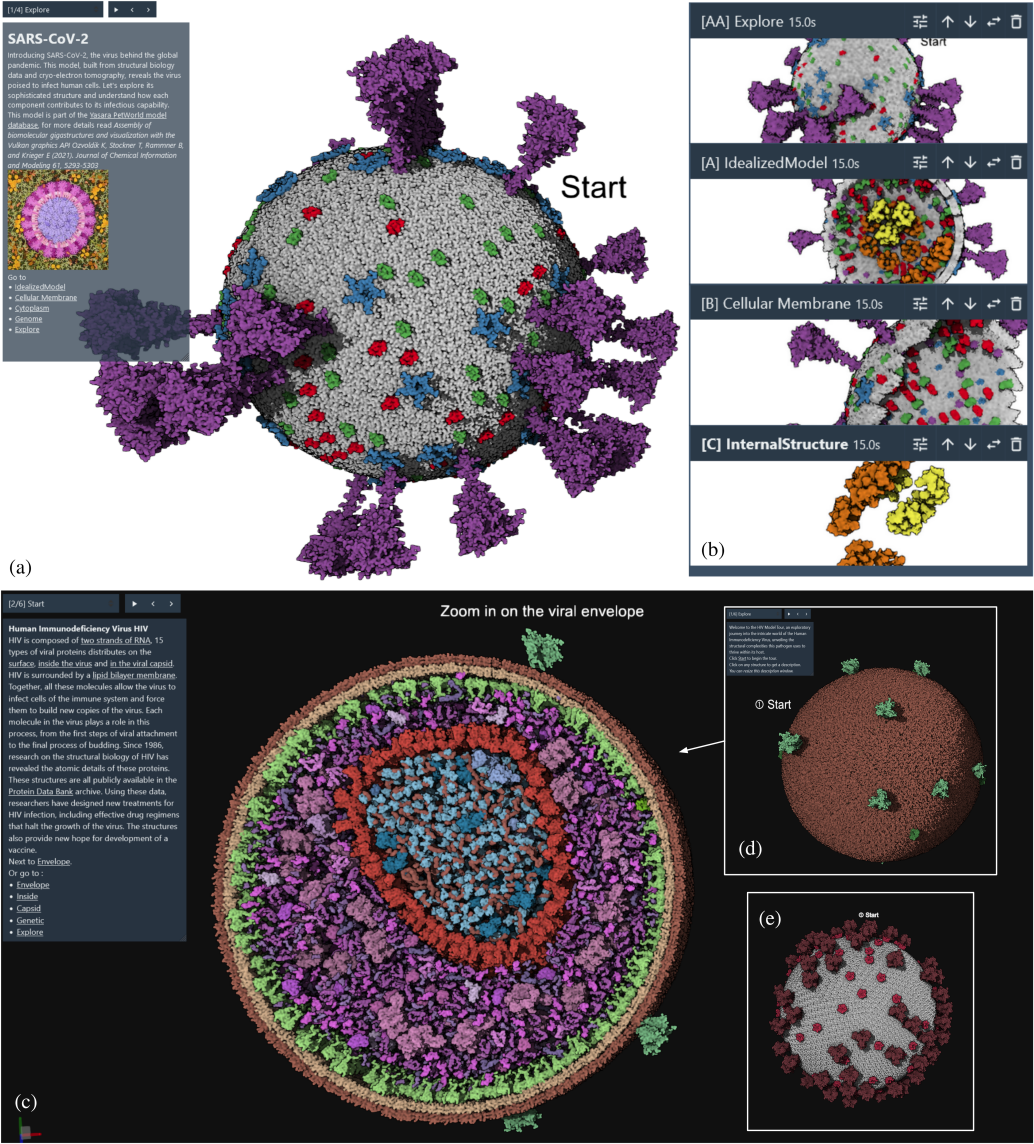
Tours of viruses. (a) Initial default view showing an overview of the entire SARS‐CoV‐2 virion. (b) Four snapshots from the tour, exploring structural aspects of the envelope and interior. (c) *cellPACK* HIV tour showing interior view and (d) starting snapshot. (e).*YASARA* PetWorld HIV tour starting snapshot.

SARS‐CoV‐2 is arguably the most recognizable virus for most of the general public since the pandemic. We used one of the currently available models developed by the *YASARA* Team (Ozvoldik et al., 2021) and plan to support alternative models made with the Mesocraft (Nguyen et al., 2021) modeling method. Being one of the smallest models, this tour, illustrated in Figure 8a,b, is easily run on any device compared to the larger billions‐atom models such as the pre‐synapse model.

In addition, two models of mature HIV virions are currently available to the community, one from the *YASARA* PetWorld database (Ozvoldik et al., 2021) and one from the *cellPACK* team (Johnson et al., 2014) (see Figure 8c–e). *Mesoscale Explorer* allows exploration of the overall similarity of the ultrastructure and components of both models, and highlights differences between them. For example, the *YASARA* model includes two genomic RNA of the virion using known secondary structure, while the *cellPACK* model is a random walk model of the two single strand gRNAs. Users can also explore differences in the distribution of Matrix protein under the lipid membrane, as well as the lipids membrane itself. *YASARA* uses a rhombus tiling approach while *cellPACK* uses a modified lipidWrapper (Durrant & Amaro, 2014) approach designed to run on GPU.

#### 3.2.2 Bacteria: mycoplasma

**FIGURE 9:**
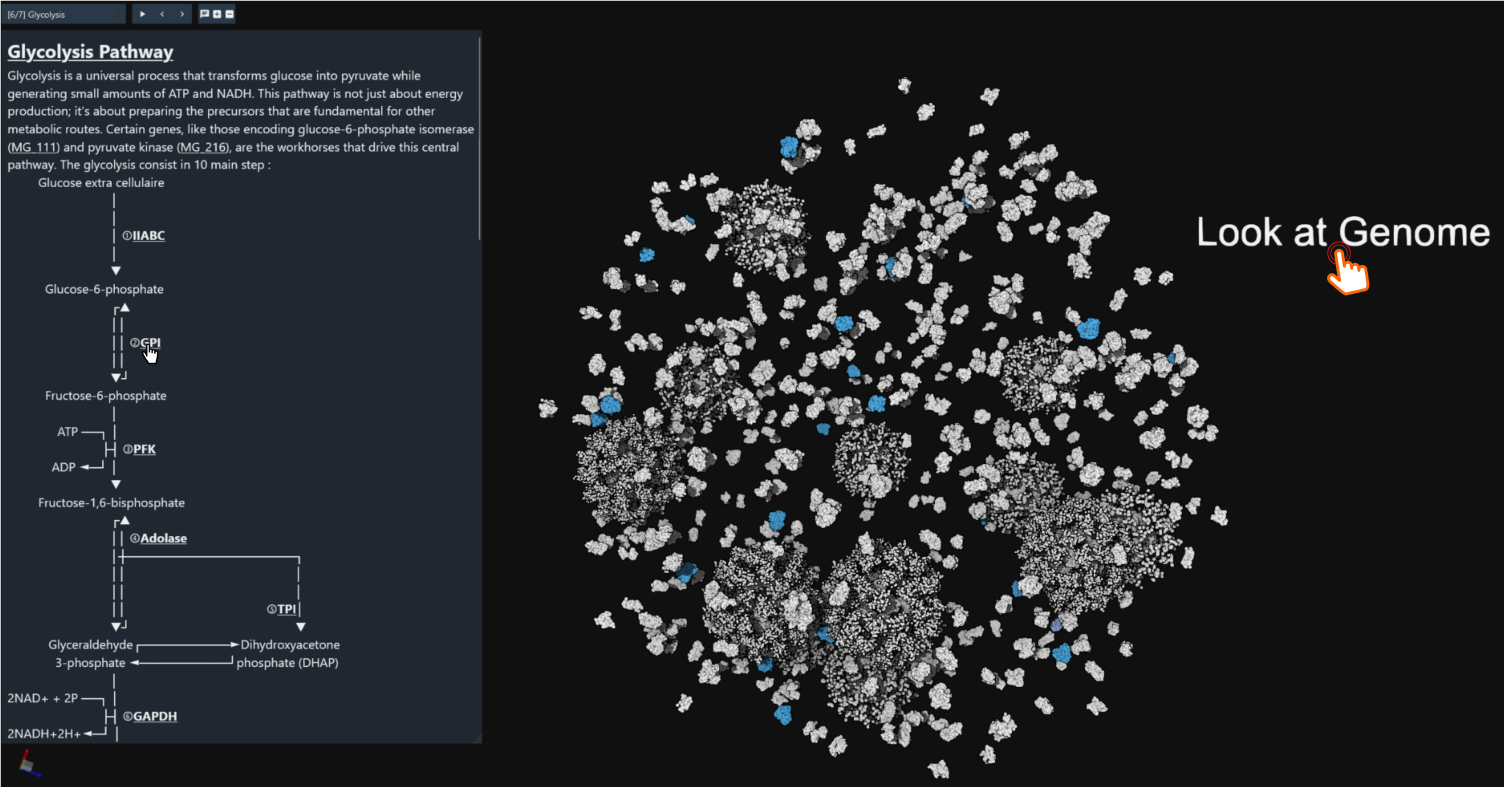
Mycoplasma tour example of guided snapshot of the glycolysis pathway. The description shows the pathway, with mouse over effects that highlight in the model the underlying enzyme. An interactable label can be seen, ‘Look at Genome’, which will bring the user to the next snapshot if clicked.

This tour is the longest, because of its complexity and the availability of a great amount of metadata. Every entry has *UniProt* (UniProt Consortium, 2023) and *KEGG* (Kanehisa et al., 2023) information. For instance, we can retrieve a pathway and design a markdown description that uses ASCII art to provide a graphical map of a reaction pathway. As shown in Figure 9, we use this approach to showcase the glycolysis pathway.

#### 3.2.3 Machinery of life

**FIGURE 10:**
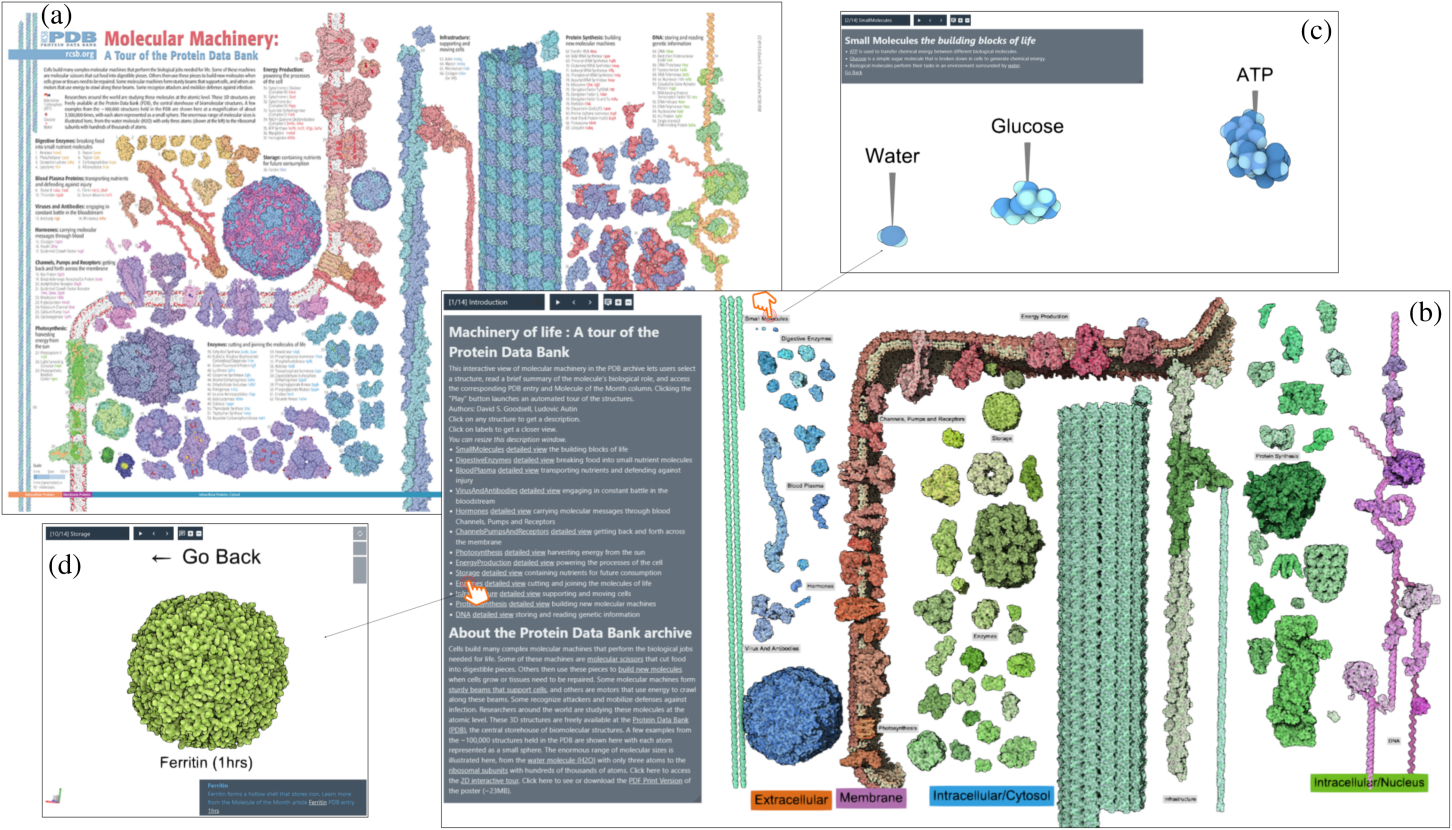
Machinery of Life tour, exploring diverse biomolecules from the Protein Data Bank archive. (a) Poster available at RCSB Protein Data Bank website. (b) Interactive version created in *Mesoscale Explorer*. (c) Detailed views of individual molecules are provided by clicking on the overall view. (d) Detailed views of the Storage category provided by clicking on the description hyperlink. Explore this interactively.

In 2002, the *RCSB PDB* published “Molecular Machinery: A Tour of the Protein Data Bank.” This illustration, available as a poster and handout, has been distributed to thousands of PDB users, teachers, and students over the years. To celebrate the release of over 100,000 structures in the PDB archive in 2014, the RCSB PDB updated this iconic image in a new edition titled “Tour of the Protein Data Bank.” This new edition is also available as a 2d interactive poster (Figure 10a). We ported it to *Mesoscale Explorer* and created a 3D interactive tour of the poster (Figure 10b). The vast range of molecular shapes and sizes in the PDB is illustrated by 96 molecular machines. The structures are depicted relative to the cellular membrane and organized into categories related to function. Using the poster, we manually placed and oriented all the given PDB IDs and grouped them using the same classification. Each snapshot corresponds to a given category and provides a labeled view of all the proteins forming the group (Figure 10c,d).

### 3.3 Future direction

Mol* is an open‐source project that is actively being developed, ensuring that the *Mesoscale Explorer* will benefit from all advancements in the core library. Moreover, now that the foundational elements are in place, we are poised to support additional file formats; to explore automated tours inspired by Molecumentary (Kouril et al., 2021; Kouril et al., 2023), to implement better story editing inspired by ScrollyVis (Mörth et al., 2023); to develop enhancements in advanced lighting effects such as global illumination; as well as supporting dynamic data similar to the *Simularium Viewer* (Lyons et al., 2022).

## 4 CONCLUSION

In our endeavor, we've struck a critical balance between rendering fidelity and efficiency by implementing strategies like level of detail (LOD) and culling. Contrary to intuition, reducing rendering fidelity can enhance perceptual clarity, minimizing visual noise and directing focus toward pivotal details. This optimization, coupled with the guided tour functionality, elevates the user experience by weaving a narrative through the intricate molecular terrains, facilitating interactive engagement with these complex structures.

The widespread accessibility of molecular visualization tools, accelerated by the COVID‐19 pandemic, has opened new avenues for researchers, educators, and the broader public to engage with molecular science. Platforms like Mesoscale Explorer, built upon the well‐tested framework of Mol*, are at the forefront of this democratization effort. The foundational data management and visualization features of Mol* are robust and stable, allowing ready prototyping of features such as LOD rendering and efficient rendering of group of spheres instances that are specific to mesoscale models and guided tours. Features developed for Mesoscale Explorer can be used in Mol*, and vice versa, ensuring a cohesive and flexible development environment.

The absence of a standardized format or repository for mesoscale models and their guided tours highlights the need for a unified system. Initiatives like MolViewSpec (Bittrich et al., 2024) propose a standard format that could be extended for such tours, while PDB‐DEV (Vallat et al., 2021) offers a potential repository for storing these models. The creation and maintenance of these standards will likely involve community‐driven efforts, focusing on licensing, contribution strategies, and iterative refinement processes.

Fueled by the exponential growth in computational power and rendering technologies, the domain of molecular visualization is advancing rapidly. This progress is not only deepening our understanding of the molecular underpinnings of life but also setting the stage for groundbreaking discoveries and applications across medicine, biotechnology, and related fields. As we continue to peel back the layers of biological complexity, we move closer to unveiling the intricate web of processes that orchestrate life itself.

## AUTHOR CONTRIBUTIONS

**Alexander Rose:** Conceptualization; methodology; software; validation; writing – review and editing; visualization. **David Sehnal:** Software; validation; methodology; writing – review and editing; conceptualization. **David S. Goodsell:** Resources; writing – review and editing; data curation; funding acquisition. **Ludovic Autin:** Writing – original draft; writing – review and editing; supervision; conceptualization; software; visualization.

## Data Availability

WebApp release and its documentation: https://molstar.org/me/. Open‐source repo on Github: https://github.com/molstar/molstar.
